# Heritability and Fitness Correlates of Personality in the Ache, a Natural-Fertility Population in Paraguay

**DOI:** 10.1371/journal.pone.0059325

**Published:** 2013-03-19

**Authors:** Drew H. Bailey, Robert S. Walker, Gregory E. Blomquist, Kim R. Hill, A. Magdalena Hurtado, David C. Geary

**Affiliations:** 1 Department of Psychology, Carnegie Mellon University, Pittsburgh, Pennsylvania, United States of America; 2 Department of Anthropology, University of Missouri, Columbia, Missouri, United States of America; 3 School of Human Evolution & Social Change, Arizona State University, Tempe, Arizona, United States of America; 4 Interdisciplinary Neuroscience Program, University of Missouri, Columbia, Missouri, United States of America; 5 Department of Psychological Sciences, University of Missouri, Columbia, Missouri, United States of America; Durham University, United Kingdom

## Abstract

The current study assessed the heritability of personality in a traditional natural-fertility population, the Ache of eastern Paraguay. Self-reports (n = 110) and other-reports (n = 66) on the commonly used Big Five Personality Inventory (i.e., extraversion, agreeableness, conscientiousness, neuroticism, openness) were collected. Self-reports did not support the Five Factor Model developed with Western samples, and did not correlate with other-reports for three of the five measured personality factors. Heritability was assessed using factors that were consistent across self- and other-reports and factors assessed using other-reports that showed reliabilities similar to those found in Western samples. Analyses of these items in combination with a multi-generation pedigree (n = 2,132) revealed heritability estimates similar to those found in most Western samples, although we were not able to separately estimate the influence of the common environment on these traits. We also assessed relations between personality and reproductive success (RS), allowing for a test of several mechanisms that might be maintaining heritable variation in personality. Phenotypic analyses, based largely on other-reports, revealed that extraverted men had higher RS than other men, but no other dimensions of personality predicted RS in either sex. Mothers with more agreeable children had more children, and parents mated assortatively on personality. Of the evolutionary processes proposed to maintain variation in personality, assortative mating, selective neutrality, and temporal variation in selection pressures received the most support. However, the current study does not rule out other processes affecting the evolution and maintenance of individual differences in human personality.

## Introduction

In Western cultures, personality is often conceptualized as varying along five dimensions: extraversion (high sociability, risk taking), agreeableness (socially cooperative, pleasant), conscientiousness (socially reliable, organized), neuroticism (emotionally unstable), and openness to experience (novelty-seeking, curiosity) [1]. Recent research on the evolution of personality takes seriously the many studies indicating that these traits show moderate heritabilities, usually between.3 and.7 [2]; studies on nonhuman animals’ personalities have yielded estimates within this range and some slightly lower [3]. One potentially serious drawback of these human studies, however, is reliance on samples from modern, Western societies. Degree of heritability has been shown to vary as a function of environment. In the cases of height and children’s intelligence, lower heritabilities have been reported in populations presumed to experience harsher environmental conditions than is typical for samples in most behavioral genetic studies [4], [5], [6]. Similar results have been shown in wild animal populations [7]. Humans lived as hunter-gatherers for much of their evolutionary history, and thus at least part our psychology is likely to have been shaped during that time. Therefore, it is critical to investigate the heritability and the reproductive consequences of personality variation among natural-fertility populations living in conditions more similar to our evolutionary past than participants in Western studies of personality. Our study of one such population, the Ache, offers a unique opportunity to assess the heritability of personality for people living in harsher conditions than people participating in Western studies, and to test hypotheses regarding the potential evolutionary significance of personality differences.

Evolutionary psychology has focused more on explaining and discovering human universals (at least within sex) than on explaining heritable individual differences [8]. Recently, however, empirical attention has turned to explaining the mechanisms through which heritable individual differences in personality can be maintained within a population. Different evolutionary mechanisms, and certainly different selection pressures, have been proposed to maintain genetic variance in different personality traits [9], [10]. Furthermore, several links have been forged between personality traits and evolutionarily relevant outcomes, such as between extraversion and number of lifetime sex partners [11], and between agreeableness and positive health outcomes [12]. However, conclusions based on these studies are again limited by reliance on modern, low fertility populations. Alvergne et al.’s [13] study of personality and RS in a Senegalese population is a notable exception. Men who were above average in extraversion attainted higher social status, were more likely to marry polygynously, and had more children than their more introverted peers. The results are consistent with positive selection for extraversion in men in this population, but overall there is little consensus as to the evolutionary mechanisms that maintain heritable variation in human personality [14].

The candidate mechanisms include (i) selective neutrality, (ii) polygenic mutation-selection balance, and (iii) balancing selection, amongst others [8], [10], [14], [15]. Table 1 shows the pattern of results that would be consistent with each of these mechanisms. For instance, polygenic mutation-selection balance predicts main effects of personality on RS and assortative mating, but not necessarily interactions for this trait with sex or the other dimensions of personality. Spatial-temporal variation, in contrast, predicts different relations between personality and RS across historical periods but not necessarily interactions between sex and personality or among different dimensions of personality.

**Table 1:** Descriptions and Predictions by Hypothesis and Actual Findings.

	Description	Relevant citations	Effect of personalityon RS	Interactions among personality traits or nonlinear relations	Personality by sex interaction	Assortative mating
Selective Neutrality	Phenotypic variation and associatedmutations do not affect fitness	[16], [17]	No	No	No	No
Polygenic Mutation Selection Balance	Many mutations with small negative effectson fitness accumulate to create heritable maladaptive variation in otherwiseadaptive phenotypes	[13], [15], [21]	Yes	No	No	Yes
Spatial/Temporal Variation	Selection pressures change across timeand place	[27]	NP	No	No	No
Heterozygote Advantage or Epistasis	The most adaptive genotype isheterozygous, or genes interactto affect fitness	[18], [28]	NP	Yes	No	No
Intralocus Sexual Conflict	One allele or combination of alleles has opposite effects on the fitness of malesand females	[8]	No	No	Yes	No
Frequency- Dependent Selection	Relation between a genotype and fitnessis moderated by the levels of thatgenotype in the population	[31], [32]	NP	No	No	No
AssortativeMating	Males and females are similar on allelesfor a trait	[33]	No	No	No	Yes
Observed PersonalityResults	(findings from current study)		Tentative Evidence	Tentative Evidence	No Evidence	Supportive Evidence

* Yes: *p*<0.05; Tentative Evidence: *p*<0.10 NP = no prediction.

Selective neutrality can maintain genetic variation when population size is high and selection is weak [16], [17], such that individual differences in personality are not related to survival or reproduction. However, selective neutrality is an unlikely mechanism because number of lifetime sex partners and lifetime RS is related to personality in some populations [11], [17], [18], as is longevity, relationship satisfaction, criminality, psychopathology, job performance, and other potentially reproductively relevant behaviors that are correlated with personality (for review, see [12].

Polygenic mutation-selection balance occurs when many mutations with small negative effects on fitness accumulate to create maladaptive heritable variation in phenotypes that are otherwise adaptive. It appears that mutation-selection balance may contribute to variation in several domains that could affect reproductive prospects, including risk for schizophrenia, autism, and cognitive disability [15], [19]. Because of possible directional selection for some personality traits, through mate choice [20] the same may be true for neuroticism and extraversion [21], as emotionally stable individuals are universally preferred as long-term mates [22], [23], and unstable individuals and highly introverted individuals are at increased risk for some pathological conditions (e.g., schizophrenia in men) associated with lower RS [12], [24], [25]. New evidence suggests that rare variants may explain most of the genetic effects on personality, and that inbreeding is associated with less socially desirable levels of personality traits, consistent with mutation-selection balance [26].

Balancing selection is most likely to occur when environments are not so stable that traits are universal, but not so variable that traits are primarily adaptively plastic [8]. Four classes of balancing selection have been proposed as potential evolutionary-genetic explanations of common, heritable variation in human personality and psychological functioning [15]: Spatial and temporal variation, heterozygote advantage, antagonistic pleiotropy, and frequency-dependent selection. Spatial and temporal variation occurs when selection pressures change across time and place such that no single phenotype has a consistent reproductive advantage (e.g., [27]). Selection can maintain genetic variation in traits for which a heterozygous phenotype is the most adaptive, and a few examples have been demonstrated (e.g., sickle-cell anemia; [28]), perhaps because selection eventually produces a solution to the problem of maladaptive homozygous phenotypes [15]. Epistasis occurs when genes moderate each others’ effects. At the phenotypic level, Eaves et al. [18] found that in postmenopausal women neuroticism and extraversion interacted to predict lifetime RS. The combinations of high neuroticism and low extraversion and high extraversion and low neuroticism were associated with the highest RS, whereas the combination of high extraversion and high neuroticism and low neuroticism and low extraversion were associated with the lowest. One result is maintenance of variation in both neuroticism and extraversion in women.

Antagonistic pleiotropy occurs when an allele affects multiple traits, and the effect on one or more of those traits is negatively related to fitness and positively related to fitness on one or more other traits [29]. The current study tests specifically for intralocus sexual conflict, which occurs when a trait has opposite effects on the fitness of males and females. Penke [8] proposed that variation in antisocial personality traits, which are more positively related to male than female RS [30], could be maintained in this way. Indeed, antisocial men who tend to be low on anxiety and high on extraversion tend to have many sexual partners, but as noted, Eaves et al. [18] found that a similar combination of traits was related to lower RS in women.

Frequency-dependent selection occurs when the relation between a heritable phenotype and fitness is moderated by the frequency of that phenotype in the population. Wilson [31] hypothesized that frequency-dependent selection would act on trait specialization, such that individuals able to adopt a social niche would exist at some equilibrium with individuals that could migrate between niches. Mealey [32] proposed a version of this hypothesis with respect to antisocial behavior (e.g., high extraversion, low conscientiousness) which may be a frequency-dependent strategy maintained at a low frequency in the population.

Assortative mating is another potential mechanism that maintains variation in human personality. If individuals mate assortatively, and if assortative mating provides a selective advantage, it can maintain variance in a population by carving out a high fitness niche for individuals at all levels of a personality trait.

Evidence from Western samples suggests that humans mate assortatively, albeit weakly (correlation coefficients between couples in a large sample ranged from 0.04 to 0.16; [33]) on several personality traits. Also, individuals with similar levels of personality traits have been shown to form higher quality relationships [34]. Alvergne and colleagues [13] reported a correlation coefficient between couples of.39 on extraversion in a high fertility population. However, reproductive decisions in humans, especially in traditional societies, are often not simply autonomous choices made by the individuals in a relationship but are regulated by parents and other kin [35], [36], [37]. Though parents’ preferences for their children’s partners’ characteristics are likely somewhat similar to their children’s own preferences, there is some evidence that preference for pleasant personality traits is weighted more heavily by children than by their parents during mate choice [38]. As a result, the extent to which the assortative mating hypothesis is a plausible explanation for the maintenance of heritable variation in human individual differences is unclear.

### Current study

The current study assessed personality in a traditional natural-fertility population, the Ache of eastern Paraguay. We used self-report methods common in Western studies for the Big Five Inventory and submitted these to confirmatory factor analysis to determine if the same five dimensions identified in Western samples emerged in the Ache. We also collected other-reports of personality for a group of men. The personality assessments combined with a multi-generational pedigree enabled estimates of the heritability of the Big Five dimensions of personality in the Ache.

The data we were able to collect did not allow us to assess all possible mechanisms that have been hypothesized to contribute to the maintenance of heritable variation in personality, but we were able to assess several of them. Interactions between personality and sex as related to RS were assessed to test the intralocus sexual conflict hypothesis. We also tested the hypothesis that assortative mating contributes to variation in personality traits. Finally, in an exploratory analysis, we investigated whether offspring personality was associated with maternal RS; specifically, by averaging sibling values on heritable personality traits and examining whether mean child personality was associated with maternal RS. If child personality influences maternal RS, then temporal variation may be contributing to the maintenance of individual differences in personality.

## Materials and Methods

### Ethics Statement

The study was reviewed and approved by the Institutional Review Board of the University of Missouri. Verbal consent was obtained from all participants.

### Participants

The Ache are indigenous to eastern Paraguay and until the 1970s were full-time hunter-gatherers [39]. Some now live on a reservation near the Mbaracayu Natural Reserve, and many of them still make excursions into the forest, during which men spend about seven hours per day hunting [40] and women spend six hours per day on work activities such as moving camp and harvesting and processing food [41]. Data were collected in two Ache communities near the Reserve: Kuetuvy (KT) and Arroyo Bandera (AB). When in their communities, individuals spend their time working in agricultural fields, on household tasks, and on watching or participating in soccer and volleyball games. Migration between these villages occurs and some individuals have close relatives living in both villages.

Participants included 77 KT residents (40 male, mean age = 33.72, SD = 13.97) and 33 AB residents (18 male, mean age = 26.97, SD = 10.27). Participants had an average of 3.55 children (SD = 3.49). After adjusting for the Lowess function of fitness by age (Supplementary Material, Fig. S1) and sex, the mean number of children did not differ across villages (*t* [105] = −0.20, *p* = 0.84; raw means = 4.03, 2.45 for KT and AB, respectively).

Heritability analyses included a sample of 2,132 people from a detailed pedigree [39]. Ache lifestyles were significantly different before contact in the 1970s, with a focus on the search for food [39]. Men provided the majority of calories, primarily from meat [42]. Hill and Hurtado described these people as “the most competent forest people we have ever seen, and the most generous in sharing their food with each other in a band-level context. Their hunting and tracking skills and arboreal agility far surpass those of any other lowland South American population we have observed (p. 78).” During this period, individuals travelled in bands characterized by flexible membership and ranging from 3 to 160 individuals in size [43]. Approximately every two or three years, up to 400 Ache individuals would gather in the forest and clear out a large space, in which the adult males would engage in long, violent, and turbulent club fights [39]. The Ache marriages were initiated primarily by courtship. Also, during this time period, the Ache believed in partible paternity, the belief that multiple men can be co-genitors of a single child.

From 1971 to the end of 1977, most Ache bands made first peaceful contacts (p. 100, [39]) with non-Ache Paraguayans and Westerners. This led to changes in social structure: Young Ache men who became the most quickly accustomed to life outside the forest by learning Spanish and non-Ache Paraguayan customs gained status and sometimes gained the wives of the older men who did not adapt as quickly. Also, virgin soil epidemics (i.e., epidemics resulting from a people’s first exposure to a new disease) killed approximately 40% of the Ache individuals during this time [39].

Beginning around 1980 through the present, Ache spend less time in the forest and instead practice subsistence horticulture, primarily manioc, which constitutes the majority of their diet [39]. Some men also participate in wage labor, but in 1992, the average Ache nuclear family’s net worth was only $12 [39]. The Ache primarily practice a flexible residence pattern with frequent spouse switching among young individuals, as before the reservation period, but do not participate in many of the ceremonies traditional to the Ache during the forest period, and no longer participate in club fights or partible paternity. Marriages are still initiated primarily by courtship. Hill and Hurtado [39] describe Ache “social and political life” as “in a state of transition … with missionary and Paraguayan peasant influence increasing over time (p. 79).”

### Measures

The commonly used Big Five Inventory was used to assess personality [1]. We used a 44-item Spanish version developed and validated by Benet-Martínez and John [44] in samples of native Spanish speakers and bilingual (English and Spanish) speakers. An Ache individual fluent in Spanish was able to translate 43 of the 44 personality items into the Ache language. Participants’ RS and genetic relatedness were determined from the pedigree. To capture individual differences beyond that associated with age-typical number of offspring, RS was operationalized as the difference between participant’s actual number of offspring and their number of offspring predicted by the Lowess function of fitness on age.

### Procedure

Individuals in both villages were informed about the study by the visiting investigators, an Ache research assistant, or by word of mouth. The testing area was a large room in a village building. The Ache translator of the personality items administered the survey to participants in groups of 2 to 11. Participants sat around a table while the interviewer described the 5-point scale used to answer each item [ranging from “describes me very well” (5) to “does not describe me at all” (1)]. Then the interviewer read each question once in Ache and once in Spanish. Participants wrote their responses on the 5 point scale onto a sheet of paper with spaces designated for each item’s answer. Thirteen individuals in KT were unable to write and thus were assessed individually by the interviewer who recorded the responses. A separate interviewer conducted the survey in AB, where participants sat at desks in a school classroom. The interviewer used the same methods as the interviewer in KT. All participants in both villages were compensated 20,000 *Guarans* (approximately $4.50).

### Reliability and Validity

Consistent with a recent study by other personality researchers in another lowland South American population [45], reverse-coded items were sometimes positively correlated with other items. After removing the reverse scored items, the α values were still generally lower than found in Western samples (extraversion:.54; agreeableness:.62; conscientiousness:.57; neuroticism:.44; openness:.72). The range of these values and the low reliability for neuroticism are consistent with the findings from this previous study [45].

A confirmatory factor analysis (CFA) was run on the 43 items to determine if the same five factor model fitted to Western samples provided an acceptable fit in this population. Preliminary analyses and inspection of the surveys indicated that many participants were not using the entire 5-point scale (i.e., some participants used only 1, 2, and 3, whereas others used 3, 4, and 5). To control for this, the first model included a method factor on which every item loaded and was constrained to be equivalent to all other loadings. For model identification, the highest loading item from each non-method factor was set to 1 (or −1, in the case of negatively loading items).

This model was highly significant (χ^2^ [849] = 1775.03, p<0.00005), indicating the five factor model was a poor fit. Other model indices also indicated poor fit (RMSEA = 0.100, TLI = 0.41). Though not desirable statistically, the RMSEA for this model was within the range of RMSEA values for many personality measures [46]; nonetheless, this model’s fit based on the TLI is substandard [Hopwood & Donnelan, [46], reported TLI values ranging from 0.52–0.70].

To test if poor reliabilities and model fit were due to poor item translation or because the five factor model is less appropriate for describing Ache individuals than individuals from Western cultures, KH, an anthropologist with over 30 years of experience working in these and other Ache communities, rated 66 Ache men on all of the survey items that had confirmatory factor analysis loadings greater than 0.4 (items appear in Table 2). Others’ reports of personality traits are underutilized and perhaps more useful in some ways than self-reports and on average yield agreement between self-reports and other reports in the range of.39 to.51 for friends and from.18 to.32 for work colleagues [47]. These reports were much more reliable than the self-reports (α values for extraversion, agreeableness, and openness were.93, .86, and .96; conscientiousness and neuroticism only contained one item each so alphas could not be calculated). Thirty-one of the men rated by KH had been administered the 43 personality items, and the remaining 35 were new. The combination of the administered items and those provided by KH increased the sample with at least self-reported or other-reported personality data to 145 individuals.

**Table 2:** Items Used to Calculate Factor Scores, with English Translations.

Extraversion (other-report; Mean = 3.28; SD = 1.31; α = .93)		
1.	tiende a ser callado **(R)**	tends to be quiet
2.	es asertivo, no teme expresar lo que quiere	is assertive, not afraid to express what one wants
Agreeableness (other-report; Mean = 3.35; SD = 1.16; α = .86)		
1.	tiende a ser criticón **(R)**	tends to be critical
2.	es considerado y amable con casi todo el mundo	is considerate and kind to almost everyone
3.	es indulgente, no le cuesta perdonar	is forgiving
Conscientiousness (self- and other-report; Mean = 3.24; SD = 1.19; α = 62)		
1.	hace planes y los sigue cuidadosamente	makes plans and follows them carefully
Neuroticism (other-report; Mean = 3.00; SD = 1.07)		
1.	se preocupa mucho por las cosas	is very concerned about things
Openness (self- and other-report; Mean = 3.38; SD = .77; α = .60)		
1.	es original, se le ocurren ideas nuevas	is original, comes up with new ideas
2.	tiene intereses muy diversos	has many diverse interests
3.	es inventivo	is inventive
4.	es ingenioso, analítico	is clever, analytic
5.	le gusta reflexionar, jugar con las ideas	likes to reflect, play with ideas
6.	es educado en arte, música, o literatura	is educated in art, music, or literature

Note: For other-reported items, α is based on the items in this table. For self- and other-reported items, α is based on the means of self- and other-reports. Neuroticism is based on only 1 item, so α is not reported. All means and standard deviations are based on a 5-point scale. R = reverse coded.

When the self-report items that loaded greater than.4 on the corresponding latent factor found in Western samples and corresponding other-report items were standardized (M = 0, SD = 1) and summed to create factor scores, only conscientiousness and openness to experience (0.45, 0.43; *p*s = 0.01, 0.02, respectively) were significantly correlated between raters (*r*s for extraversion, agreeableness, and neuroticism = 0.00, 0.03, −0.07, respectively, all p>0.70). Agreements for ratings of conscientiousness and openness to experience were at the high ends of what Connelly and Ones [47] observed in a meta-analysis of correlations between self- and other-reports of personality data. Conscientiousness and openness to experience values for all 145 individuals were defined as the sum of each participant’s self- and other-report *z*-scores for each factor; data that were missing for either self (n = 35) or other (n = 72) report scores (but not both) were estimated as the average of 5 iterations of Multiple Imputation in IBM SPSS Statistics ver. 19.0. Extraversion, agreeableness, and neuroticism were defined only as the other-reports of these variables; individuals with only self-report data were not included in analyses involving these factors (n = 79).

In all, the personality variables used to test evolutionary models were conscientiousness, openness to experience (the sums of self- and other-reports, n = 145), extraversion, agreeableness, and neuroticism (other-reports of men only, n = 66).

### Statistical Analyses

We calculated heritabilities for personality dimensions with their associated standard errors using an animal model commonly used for pedigree analyses [48], [49]. The same set of fixed effects (sex, village, and linear effect of age) and random effects (breeding value and residual) was used for all five personality variables. The covariance matrix for the breeding values was assumed to be **A** with elements 2θ_ij_V_A_ with kinship coefficients (for individuals i and j as θ_ij_) calculated from an Ache pedigree of 2,132 people. Residuals were assumed to be uncorrelated (**I**V_R_). All quantitative genetic models were run in WOMBAT [50]. Advantages of the animal model include no assumptions associated with assortative mating or inbreeding [49]. However, the current study lacked a measure of common environment, which would lead to the overestimation of the heritability of personality traits if common environment contributes to personality [49]. Generally, however, common environmental influences have been shown to be small in studies of personality using twin, family, and adoption designs [2].

Then, we regressed RS on personality. We then separately investigated the moderating effects of sex and the other personality variables by separately adding their interactions to the initial model. Assortative mating was assessed by calculating correlations between spouses.

To assess the current study’s power to detect relations between personality and RS, we used the observed relation between extraversion and RS (i.e.,.27) in the study that is most similar to the current study [13] as the target effect size. However, the reliability of the extraversion measure used in that study was.55, so assuming there was no error in the measure of RS, the correlation between extraversion and RS, corrected for attenuation, was.27/(.55^.5^), or.36. By multiplying this value by the square root of the reliability for each measure in the current study (Table 2), we calculated the expected observed correlation in our sample, assuming a true correlation of.36. Based on this value and the sample sizes for each trait, the two-sided power to detect an effect of this size is.86 for extraversion, .82 for agreeableness .94 for conscientiousness, and .93 for openness to experience. Because there was only one item measuring neuroticism, the reliability of the measure is unknown and the power to detect a relation between neuroticism and RS is thus unclear. The current study is likely underpowered to detect a relation between neuroticism and RS, though, given only 1 item and the lowest sample size (n = 66) of all of the measures. Also, the current study is likely underpowered to detect relations smaller than the relation between extraversion and RS obtained by Alvergne and colleagues [13].

To test whether the previous generation showed different relations between personality and RS, we first assessed which personality traits showed the highest correlations among siblings. For these traits, we averaged the personality values for siblings coming from the same mother to estimate what the mother’s personality phenotype may have been. We then regressed the estimate of maternal personality on lifetime RS. This was not done for fathers, because maternity is more certain than paternity and because maternal and paternal RS are not independent.

## Results

### Heritability

Heritability estimates (with standard errors and sample sizes) for the two personality factors based on self-reports and other-reports were.26 (.17, n = 145) and.09 (.15, n = 145) for conscientiousness and openness, respectively. For the three personality factors based only on other-reports, heritabilities were.79 (.28, n = 73),.66 (.31, n = 71), and.57 (.36, n = 66) for extraversion, agreeableness, and neuroticism, respectively.

### Personality and RS

Summary results are in Table 1, and in greater detail in Tables 3 and 4. Agreement between self- and other-report was obtained for conscientiousness and openness to experience. In models that controlled for age and sex, neither conscientiousness nor openness to experience was related to RS (ps >.80); the interactions of sex with conscientiousness and openness to experience were also non-significant (ps >.10). For these variables, no evidence was found for either mutation-selection balance (no correlations with RS) or intralocus sexual conflict (the relation did not differ by sex).

**Table 3:** Support for Different Results by Variable.

	Potential Moderators of the Relation Between Personality and Reproductive Success (RS)			
Personality Dimension	None	Non-linear relationsand RS	Sex	Assortative Mating
Extraversion	Possibly Yes*	No	N/A	N/A
Neuroticism	No	No	N/A	N/A
Agreeableness	No	No	N/A	N/A
Conscientiousness	No	Possibly Yes	No	Yes
Openness	No	No	No	Yes
Significant effects/Tests	0/5	0/5	0/2	2/2

Note: Supportive: *p*<0.05 (one-tailed for Extraversion); Tentative: *p*<0.10.

* If the analysis is a one-tailed test, this finding is statistically significant (*p*<.05).

**Table 4:** Reproductive Success Residuals Regressed on Personality.

Sex and Age Controlled	Estimates (S.E.)				
O	−.01 (.09)	.	.	.	.
Sex	−.24* (.09)	−.24 (.09)	.	.	.
Age	.08 (.09)	.08 (.09)	.09 (.12)	.07 (.12)	.06 (.13)
C	.	−.01 (.09)	.	.	.
E	.	.	.22 (.12)	.	.
A	.	.	.	.04 (.12)	.
N	.	.	.	.	−.03 (.13)
N	143	143	73	71	66
R^2^	.05	.05	.05	.01	.01
AIC	407.30	407.30	210.35	208.09	193.96
**Moderator Models**	**Estimates (S.E.)**				
O	−.04 (.09)	.	.	−.01 (.09)	−.03 (.13)
Sex	−.22* (.09)	−.23* (.09)	−.23* (.09)	−.24* (.09)	−.23* (.09)
Age	.10 (.09)	.09 (.09)	.11 (.09)	.08 (.09)	.10 (.09)
O*Sex	.15 (.09)	.	.	.	.
C	.	−.03 (.09)	.00 (.09)	.	.02 (.13)
C*Sex	.	.14 (.09)	.	.	.
C^2^	.	.	−.16 (.08)	.	.
O^2^	.	.	.	−.02 (.08)	.
O*C	.	.	.	.	−.11 (.08)
N	143	143	143	143	143
R^2^	.07	.07	.08	.05	.07
AIC	406.60	406.73	405.64	409.24	409.02

Note: E = extraversion, A = agreeableness, C = conscientiousness, N = neuroticism, O = openness to experience; all regression weights are standardized.

The interaction between conscientiousness and openness to experience, controlling for age and sex, did not correlate with RS (p = .14). There was a trend for a nonlinear relation between conscientiousness and RS, controlling for age and sex (t [138] = −1.89, p = .06), but no such relation between openness to experience and RS. Individuals with very low or high levels of conscientiousness had slightly lower RS than individuals who were average.

There were 33 male and female pairs in the pedigree with non-missing personality data that had reproduced. These couples showed strong assortative mating for both conscientiousness (r = 0.41, p* = *.02, n = 33) and openness to experience (r = 0.43, p = .01, n = 33; Fig. S2). Data on the three other traits were only available for males, as they were other-reports. To test whether partners’ personalities converge as they remain in romantic relationships (which would raise the possibility that personality similarity is not present at the start of a relationship), we regressed male personality on female personality, year of the birth of the couple’s first child, and the interaction between female personality and year of first birth. If partners’ personalities converge over time, female personality should be less predictive of male personality for couples with later years of first births, and the interaction should be significantly negative. For conscientiousness, the interaction of female conscientiousness and year of the couple’s first birth was not significant (t [29] = −1.03, p = .31). For openness to experience, the interaction of female openness and year of the couple’s first birth was a trend for statistical significance (t [29] = −2.04, p = .051), consistent with the possibility that couples’ personalities, at least openness to new experiences, converge over time.

### Other-Report for Male Personality

**Figure 1:**
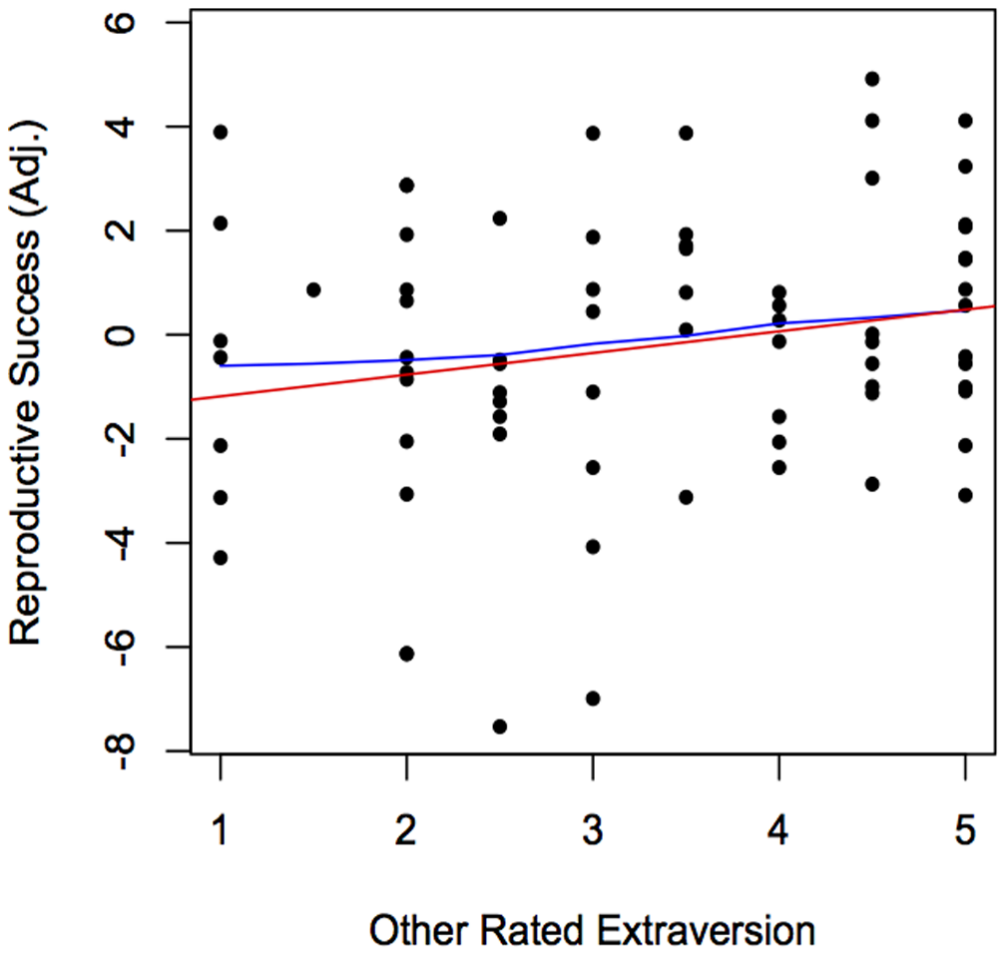
Extraversion and Male RS. The red line is based on the regression of RS on other rated extraversion; the blue curve is the Lowess approximation of the function.

Recall, these data on extraversion, agreeableness, and neuroticism included only males (see Methods). For these males, controlling for age, there was no relation between agreeableness or neuroticism and RS, but a trend for higher extraversion to be associated with higher RS (t [70] = 1.86, p = .07; Fig. 1). The estimate here is a conservative two-tailed test and if we consider the relation between extraversion and RS in men as an *a priori* attempt to replicate Alvergne et al. (9), the effect is significant (p = .035) in a one-tailed test. Separate models controlling for age, sex, and linear effects of extraversion (model 1), agreeableness (model 2), or neuroticism (model 3) yielded nonsignificant non-linear relations between each of these personality variables and RS (Table 4).

### Maternal Personality

Agreeableness, conscientiousness, and openness were the only variables with maternal sibling intraclass correlation coefficients greater than 0 (ICCs = .41, .21, and.22, respectively). To verify that these values do not simply reflect environmental effects of having few or many siblings, the ICCs were re-calculated after adjusting for number of maternal siblings. After this, only agreeableness (ICC = .35) and conscientiousness (ICC = .13) had positive ICCs. We then assessed the relations between these variables and maternal RS using Poisson regression, with RS operationalized as lifetime number of children that survived to age 5 or older. There was a significant positive relation such that the higher the average child agreeableness the higher the average maternal RS (n = 56, z = 2.86, p* = *.004), but there was no relation between average child conscientiousness and maternal RS (n = 80, z = 1.24, p* = *.22).

## Discussion

The current study assessed personality in a traditional natural-fertility population, the Ache of eastern Paraguay, using the standard Big Five model developed in Western samples. We provide the first assessment of the heritability of these five dimensions of personality (i.e., extraversion, agreeableness, conscientiousness, neuroticism, openness) in a traditional natural-fertility population. Moreover, by examining the relations between individual differences in personality and reproductive success we were able to test several mechanisms have been proposed to explain the maintenance of genetic variation in human personality [14]; specifically, intralocus sexual conflict, assortative mating, selective neutrality, and temporal variation in selection pressures.

### Personality Assessment

The five-factor model found for Western populations was a poor fit to the self-reported personality data in the Ache. A comparison of other-reports to self-reported data indicated that two factors, openness to experience and conscientiousness, were measured consistently across raters, whereas the other personality dimensions showed no agreement between self- and other-reports. Because we did not collect other types of reliability data (e.g., test-retest reliability, other-reports from other Ache individuals), it is unclear whether the five-factor model was a poor fit because the Ache have different personality configurations than other populations, because of issues with the translation of the items, or because of issues with the 5 point scale on which participants rated items. Based on the problems with reverse-coded items found here and in another recent study in an indigenous South American population [45], we suspect the latter is most probable.

### Heritability

The heritabilities we observed fluctuated from values lower than the range usually observed in Western societies to higher values. However, taken together, these values indicate that the hypothesis that heritability of personality is significantly lower in this population than is found in Western samples is not strongly supported, although our estimates should only be considered suggestive at this point given their large standard errors. Across personality tests, openness to experience was the least heritable factor, and extraversion was the most heritable. Whether this variation across personality traits is an important trend in small-scale societies or random error will be better understood with future research in traditional societies.

The traits measured by other-report alone showed higher heritabilities than those assessed by self-report. If KH unconsciously used familial information to make judgments about individuals’ personalities, this would have inflated these estimates. However, KH only rated individuals he knows or knew well. Additionally, KH’s ratings showed much higher reliabilities than the self-reports, and observer reports have previously been shown to result in higher correlations between relatives on personality measures [51], which could also account for larger heritabilities for these traits. Finally, openness may have been based on items that, among the Ache, were endorsed by individuals who had experience with Western ideas, which may be a large environmental influence that would lower heritability of openness among the Ache.

Also, the structure of our data did not allow us to separate effects of heritability and common environmental influences. The data spanned many related individuals varying greatly in both genetic relatedness and, likely, in their common environmental experiences. Though in Western samples, the measured influence of common environmental influences on personality tends to be small, it is possible that they are larger in the Ache, and that the actual heritabilities of personality in the Ache are lower than reported here.

Because of recent demographic events experienced by the Ache, including many deaths due to disease and a transition from forest to reservation life, the findings we obtained may not generalize to other small-scale societies. That said, significant demographic events are not uncommon in small-scale societies in modern times. It is possible that these events, especially the virgin soil epidemic, resulted in very high selection for some traits, such as conscientiousness, and against others, such as extraversion. Future research in small-scale societies might take advantage of available genealogical data, asking participants to rate deceased individuals from previous and current generations, and comparing the levels and heritabilities of such traits across generations. Findings from the current study, specifically that agreeableness may have been positively associated with reproductive success in the previous generation of Ache, suggest that such investigations could provide interesting new findings.

Finally, some participants in the current study were born during a time when the Ache believed in partible paternity. The attribution of biological paternity may still have been accurate during that time period [39]. Nevertheless, it is possible that the heritability estimates we presented may be slightly downwardly biased if some of the paternity assignments in the genealogy were incorrect.

### Personality and Reproductive Success

Our finding that men above average in extraversion had higher reproductive success than their peers confirms the findings of Alvergne and colleagues [13] for a Senegalese population and similar relations found in Western samples [11]. One standard deviation increase in extraversion was associated with an increase of.57 in reproductive success. Overall, however, evidence for most of the evolutionary mechanisms tested in the current study was generally weak; of the 14 statistical tests 2 yielded significant results (expected by chance = 0.7), and 4 total yielded significant or marginally significant (i.e., p<.10) results (expected by chance = 1.4, Table 3). When restricted to fitness related statistical tests only, 0 out of 12 tests yielded significant results (expected by chance = 0.6), and 2 yielded significant or marginally significant results (expected by chance = 1.2).

The relation between extraversion and reproductive success found in men (ß = .23, Table 4) was comparable in size to that observed between reproductive success and extraversion in the previous study most similar to the current study, i.e., that of Alvergne and colleagues [13]. However, other personality traits did not show substantial relations with reproductive success. These findings may generalize to other traditional societies, or could be specific to the Ache. Desirable mates among the Ache may be selected on the basis of other skills, such as physical characteristics or hunting prowess. An additional possibility is that reproductive leveling, which is common in Amazonian societies because a lack of important heritable resources, such as land and livestock [52], lowers selection in the Ache for many mating-relevant traits, including personality.

Despite our replication of Alvergne and colleagues’ [9] finding of a relation between extraversion and reproductive success, we note a caution with here. The current study used an other-report method to assess extraversion. Because the rater was only rating individuals who he knows, it is possible that he unconsciously used reproductive success as a proxy for the extraversion items.

### Assortative Mating

Of the two personality factors on which data were available for both sexes, that is, conscientiousness and openness, Ache individuals mated assortatively on both. These correlations, 0.41 and 0.43, are larger than correlations between couples in Western societies [33], but similar to findings by Alvergne and colleagues [13]. The exact processes underlying high assortative mating on these aspects of personality is unclear and warrants further investigation, especially given it has now been found in two traditional, high fertility populations. One potential explanation is that individuals with similar levels of personality traits form higher quality and longer lasting relationships [34]. In this case, being similar to one’s partner on personality could increase one’s reproductive success, regardless of the individual level effects of personality, and a preference for similarity would be selected. This process would maintain individual differences in personality.

### Child Personality

Although not part of the original goals of the study, the data we collected allowed for an exploratory assessment of the relation between child personality and maternal reproductive success. The finding that mothers with children who were high in agreeableness had more children should be interpreted cautiously, but merits follow up investigation. One possibility is that agreeableness was under positive selection during forest life. Another possibility is that, for whatever reason, these children make fewer demands on parental investment than less agreeable children, which in turn may contribute to maternal reproductive success. Whatever the process, the finding suggests temporal variation in the selection pressures acting on agreeableness.

### Limitations and Future Directions

The current study suffered from some important limitations. First, the phenotypic sample size was small. Though the study was sufficiently powered to detect a relation between personality and reproductive success of the magnitude found by Alvergne and colleagues [9] for extraversion, it was too small to detect smaller but still potentially evolutionarily significant relations between personality and reproductive success. Also, as a result, the current study was not able to measure relations between personality and reproductive success at the genetic level, which is unfortunate, as genetic correlations can provide evidence about which evolutionary processes cause variation in a trait [53]. Second, three of the Big Five personality traits, as defined in Western samples, were not measured reliably across raters using self-report, and the other-reports were only available for men. As a result we were not able to assess whether the relations between these latter traits (i.e., extraversion, agreeableness, and neuroticism) and reproductive success varied by sex.

The finding that average child agreeableness was related to maternal reproductive success should be judiciously interpreted because the nature of the relation cannot be determined from these data. Because children’s personality genes come from both parents, and because personality is highly correlated between spouses, we cannot assess whether the relation between agreeableness and reproductive success holds true in mothers only, fathers only, or both.

Future studies should continue to assess construct validity when measuring personality and attempt to use genetically-informed analyses. Using larger samples would allow for the measurement of the genetic covariance between personality and reproductive success, and would increase the probability of finding smaller, potentially evolutionarily relevant, effects. Also, future studies, when possible, should use samples with only individuals at ages at which they are unlikely to continue reproducing, or should measure personality in young people and wait to measure reproductive success until they are much older, which could allow for a test of the antagonistic pleiotropy hypothesis of genetic variation in personality, namely that some traits increase early reproductive success at the cost of later reproductive success.

The relations between men’s extraversion and reproductive success and between couples’ personality scores show agreement across studies and warrant future investigation. Specifically, it is important to know whether the correlation between extraversion and reproductive success is at the genetic level or the environmental level. The former would be consistent with mutation-selection balance, whereas the latter would be interesting but would not bear on the maintenance of heritable variation in personality. The assortative mating findings warrant future investigation as well. Specifically, it would be most helpful to test for assortative mating on personality in a prospective study, to ensure that the correlations between couples’ personality traits do not only arise after they are coupled. If so, assortative mating is not likely to explain the maintenance of heritable variation in personality.

In summary, despite lingering questions, the current study is the first to measure the heritability of personality in a traditional society. We found support for the hypothesis that personality is moderately heritable among the Ache, as it is in Western societies. We found mixed support for evolutionary models of the maintenance of heritable personality variation, with selective neutrality, assortative mating, and temporal variation in selection pressures receiving the most support. Further research on this topic will continue to narrow down the possible combinations of evolutionary mechanisms that maintain heritable variation in human personality.

## Supporting Information

Figure S1 **Lowess Function of Reproductive Success by Age.** This Lowess curve (tension = 2/3) is a more flexible way to account for nonlinear variation than including quadratic components in regression analyses; however, the interpretation of results below did not differ if quadratic components rather than the Lowess values were used as controls.
(TIFF)

Figure S2 **Assortative Mating. Both of these relations remain significant after controlling for age.** Also, after simultaneously removing the two couples in the sample for which both members were interviewed by the research assistant, both relations remained significant.
(TIFF)
