# Supplementary figures and images for: Heritability and Fitness Correlates of Personality in the Ache, a Natural-Fertility Population in Paraguay
Source: PLoS One. 2013 Mar 19;8(3):e59325. doi: 10.1371/journal.pone.0059325 (PMC3602062; doi:10.1371/journal.pone.0059325)

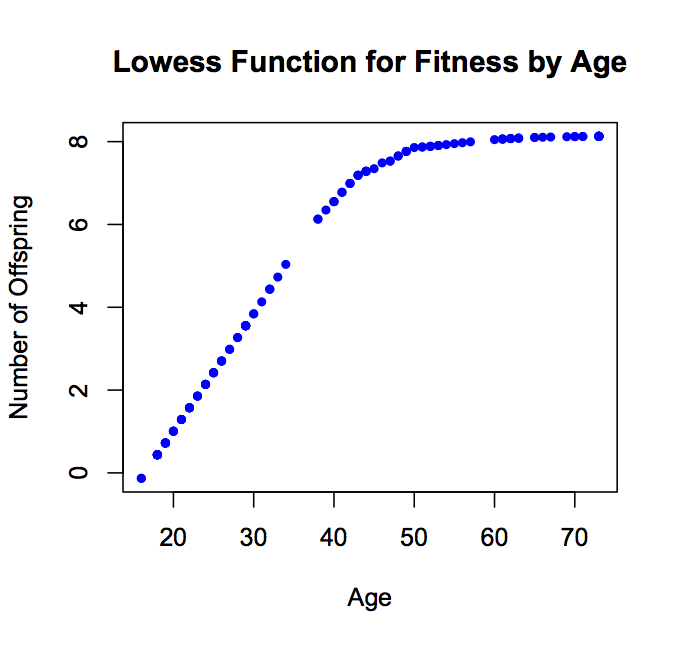

Supplement: Figure S1 — Lowess Function of Reproductive Success by Age. This Lowess curve (tension = 2/3) is a more flexible way to account for nonlinear variation than including quadratic components in regression analyses; however, the interpretation of results below did not differ if quadratic components rather than the Lowess values were used as controls. (TIFF) [file pone.0059325.s001.tiff]

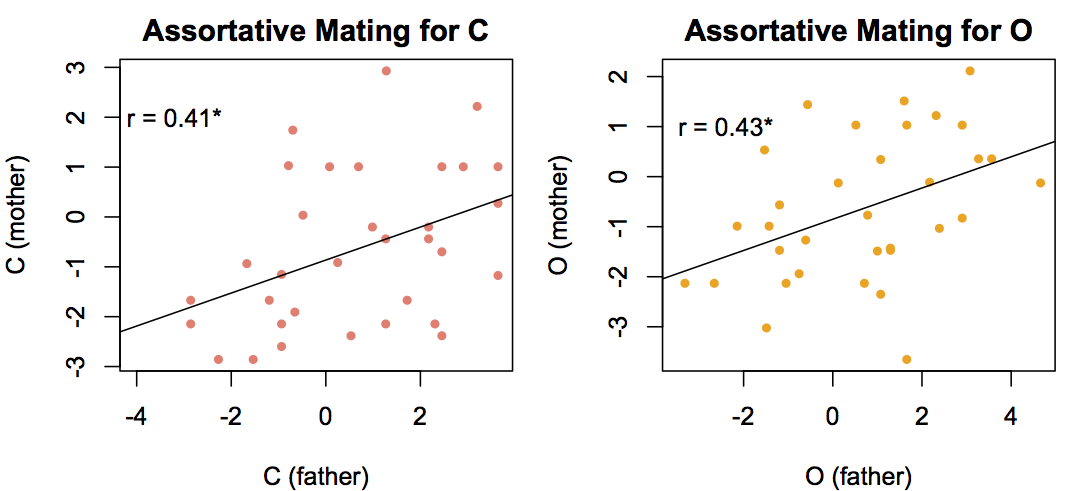

Supplement: Figure S2 — Assortative Mating. Both of these relations remain significant after controlling for age. Also, after simultaneously removing the two couples in the sample for which both members were interviewed by the research assistant, both relations remained significant. (TIFF) [file pone.0059325.s002.tiff]
